# When herpes simplex virus encephalitis meets antiviral innate immunity

**DOI:** 10.3389/fimmu.2023.1118236

**Published:** 2023-01-20

**Authors:** Linhai Zhang, Lijia Zhang, Fangjing Li, Wanyu Liu, Zhenzhen Tai, Juan Yang, Haiqing Zhang, Jinmei Tuo, Changyin Yu, Zucai Xu

**Affiliations:** ^1^ Department of Neurology, Affiliated Hospital of Zunyi Medical University, Zunyi, China; ^2^ The Collaborative Innovation Center of Tissue Damage Repair and Regeneration Medicine of Zunyi Medical University, Zunyi, China

**Keywords:** herpes simplex virus, autoimmune encephalitis, innate immune, NMDAR encephalitis, immunotherapy

## Abstract

Herpes simplex virus (HSV) is the most common pathogen of infectious encephalitis, accounting for nearly half of the confirmed cases of encephalitis. Its clinical symptoms are often atypical. HSV PCR in cerebrospinal fluid is helpful for diagnosis, and the prognosis is usually satisfactory after regular antiviral treatment. Interestingly, some patients with recurrent encephalitis have little antiviral effect. HSV PCR in cerebrospinal fluid is negative, but glucocorticoid has a significant effect after treatment. Specific antibodies, such as the NMDA receptor antibody, the GABA receptor antibody, and even some unknown antibodies, can be isolated from cerebrospinal fluid, proving that the immune system contributes to recurrent encephalitis, but the specific mechanism is still unclear. Based on recent studies, we attempt to summarize the relationship between herpes simplex encephalitis and innate immunity, providing more clues for researchers to explore this field further.

## 1 Introduction

Viral infection causes encephalitis, an inflammation of the brain parenchyma accompanied by neurological dysfunction (1). Symptoms include headache, altered consciousness, seizures, focal dysfunction, papilledema, fever, myalgia, and respiratory or digestive symptoms (2). In general, the prognosis of viral encephalitis is determined by the pathogen and host immune status, but in a small number of cases, viral infection can lead to antibody-mediated autoimmune encephalitis (AE) (3, 4). Several neurological autoimmune diseases can be induced by HSV infection in individuals with selective innate immunodeficiency (5). Unlike adaptive immunity, innate immunity cannot establish and maintain immune memory against reinfection. To restrict viral infections, antiviral innate immunity acts in a non-specific manner when the body is exposed to pathogens (6), which has recently been challenged (7). There is extensive literature claiming that the innate immune system can create memory after infection and therefore be able to respond rapidly in the event of a second infection (8, 9), while pattern recognition receptors (PRRs) are a prerequisite for this ability, PRRs may also be key signals in the induction of autoimmune encephalitis (10). The purpose of this review was to identify the possible relationship between innate immunity and herpes simplex viral encephalitis (HSVE) and to offer new insight for clinical investigation.

## 2 Viral replication

**Figure 1:**
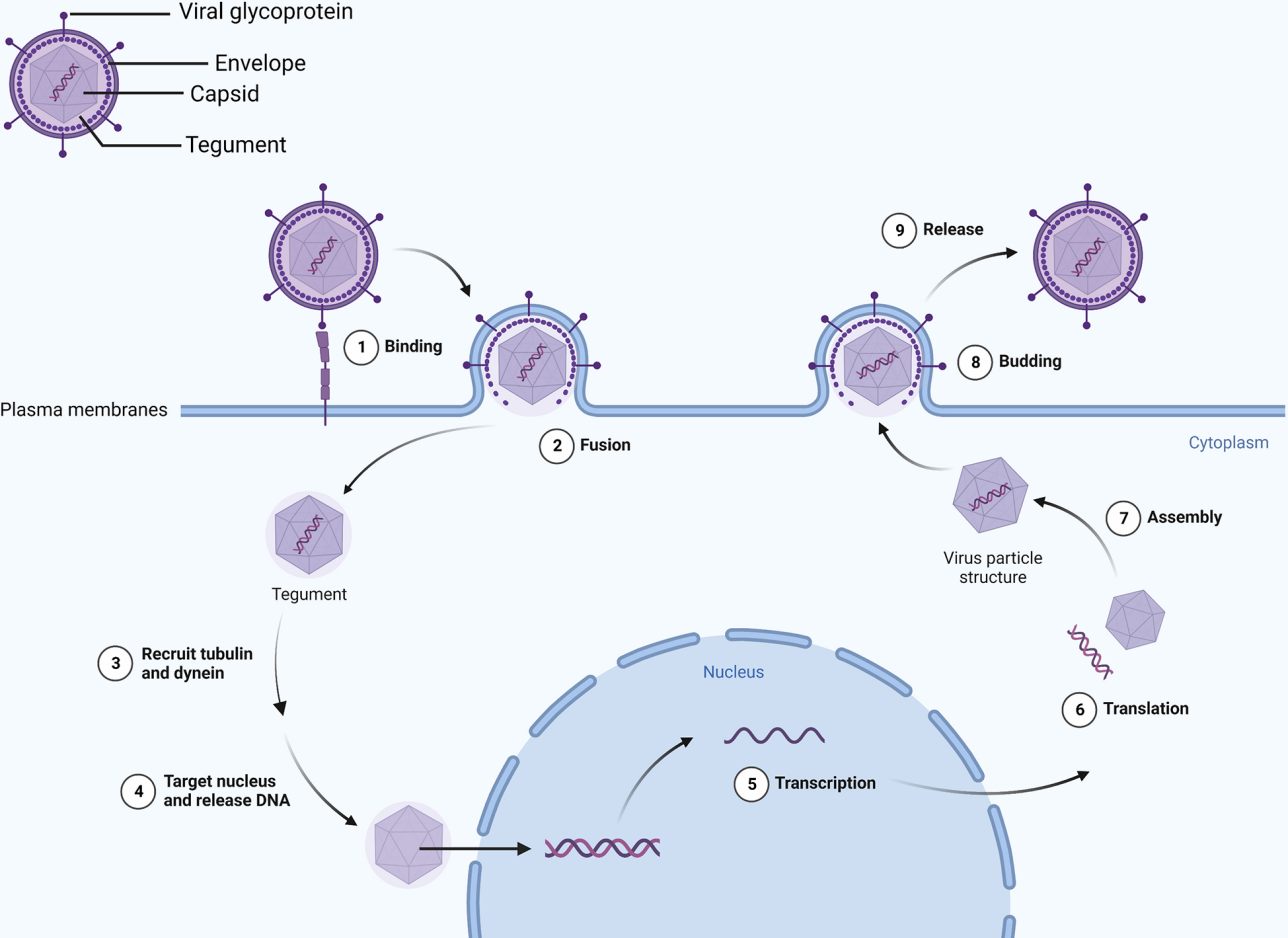
Viral envelope glycoproteins mediate binding with the cell membrane. Viral DNA enters the nucleus and transcribes and translates viral particle accessories as the envelope merges with the cell membrane. Eventually, intact virions leave the cell by exocytosis.

An HSV-1 virus consists of a capsid, tegument, and envelope in a spherical shape (11). In addition to gD, gH, gL, and gB, the envelope contains 11 viral glycoproteins, among which the gB function as a fusogen to allow HSV to enter cells. It combined with heparan sulfate, herpesvirus entry mediator, and nectin on the surface of the host cell when cells are infected with HSV using the fusion mechanism involving gB, gD, gH/gL as the core (12, 13). The tegument and capsid enter the host cell after fusion, and the tegument recruits tubulin and dynein to transport the capsid to the nucleus (14, 15). Researchers have shown that the tegument proteins UL36 and UL37 trigger movement to the nucleus (16), releasing the genome into the nucleus (17). In addition, vp16 separates from the capsid and enters the nucleus to form a complex with host cell factor 1 (HCF-1) and octamer binding protein-1 (Oct-1), and finally, it binds to the promoter of the Immediate early (IE) gene, which drives gene expression (IE, E and L genes) (18, 19). HSV codes ICP0, ICP4, ICP22, ICP27, and ICP47 (20) genes can be activated or inhibited by them, thereby promoting or delaying a process. Ultimately, the IE gene produces proteins that regulate viral replication and cellular antigen presentation, and the E gene synthesizes viral DNA and packages proteins, the L gene produces proteins for virion assembly, and mature viruses exit the cell by exocytosis (21) (**Figure 1**).

Through reverse axoplasmic transport, the virus in the exposed area enters nerve endings and reaches the neuronal cell body after lytic infection (22). Although it has been reported in the vagus nerve and superior cervical ganglion, it is also usually latent in the trigeminal ganglion (23). IE, E, and L genes start to express approximately 24-72 h after infection. IE and E transcription decreases while latency-associated transcript (LAT) gradually increases, thus forming latent infection (21). The mechanism may be related to promoting viral genome silencing by LAT (24). Local latent viruses can be reactivated and replicated by fever, emotional or hormonal imbalance, trauma, or immunosuppression and locally produce blisters, sores, or ulcers (25, 26).

## 3 Immune responses

### 3.1 Primary immune response in HSVE

For a virus to enter the brain, it must cross the blood-brain barrier, which is different from a peripheral infection. Transcellular transport is severely limited by the tight junctions between cells within the blood-brain barrier, which separates the central nervous system from peripheral blood circulation (27). However, HSV can be transported reversely along the nerve, bypassing the blood-brain barrier and entering the central nervous system, activating innate immune cells and generating an innate immune response (28), Its viral genomic DNA and some RNA intermediates become the true pathogen-related molecular patterns (PAMPs) of pattern recognition receptors (PRRs).

After the virus enters the brain, nucleic acid sensing is important to detect the virus. Microglia express cyclic-GMP-AMP synthase (cGAS), a nucleotidyl transferase and an important cytoplasmic sensor that recognizes DNA ligands in different cell types (29, 30).

Compared to wild-type mice, cGAS- and STING-deficient mice had significantly higher viral loads in brain tissue, according to Reinert et al. (29). cGAS is activated after binding to viral double-stranded DNA (dsDNA) and utilizes ATP and GTP to form cyclic GMP-AMP (cGAMP). CGAMP further activates the stimulator of interferon gene (STING), which is transported to the Golgi by COPII (31), activation of TRAF family member-associated NF-κB activator (TANK)-binding kinase 1 (TBK1) after palmitoylation, leading to the activation of interferon regulatory factor 3 (IRF3) and interferon production (32, 33). A zebrafish model of HSV-1 infection induces robust interferon production and depends on STING expression, but cGAS seems dispensable for the STING signaling, whereas DDX41 and DHX9 were found to be more closely related to interferon production in zebrafish (34).

An immune response is triggered by the DNA-dependent activator of IFN-regulatory factors (DAI), a recently discovered DNA sensor that detects nucleic acids exposed during cell damage or infection. Using artificially induced DAI and B-DNA stimulation of L929 cells, IFN was found to be expressed earlier and at higher levels than controls, which was associated with the synergy of IRF3 and TBK1 and independent of TLR9 (35). Thanh et al. demonstrated that in DAI knockdown HepG2 cells, HSV-1 viral gene and ICP0 expression were increased, but DAI knockdown did not affect cytoplasmic DNA stimulation-mediated interferon release, suggesting that there may be other pathways that can promote interferon expression (36). The receptor-interacting protein 1 (RIP1) and the receptor-interacting protein 3 (RIP3) can also be recruited by DAI through its receptor-interacting protein homotypic interaction motifs (RHIMs), then activating NF-κB (37). A new DNA sensor, IFI16, has been discovered in the cytoplasm, similar to DAI. It is a member of the PYHIN protein family with two DNA-binding domains that can directly bind to viral DNA and recruit STING (38). A nuclear localization signal allows IFI16 to recognize HSV DNA and acetylate itself, which recruits STING and induces the production of IFN (39, 40).

A cytoplasmic-localized RNA sensor, Retinoic acid-inducible gene (RIG)-I-like receptor (RLR) includes RIG-I, MDA5, and LGP2, whose enhanced expression is induced by viral infections and interferon stimulation, which leads to antiviral effects (41–43). HSV replication in mutated human hepatoma cells inactivated by RIG-I demonstrates their relationship (44). According to Emma et al., RIG-I expression is parallel with intracellular DNA load, and RIG-I cooperates with DAI to exert an antiviral effect on HSV through RNA polymerase 3 (45), it is unclear, however, whether RIG-I recognizes RNA transcribed by HSV.

**Figure 2:**
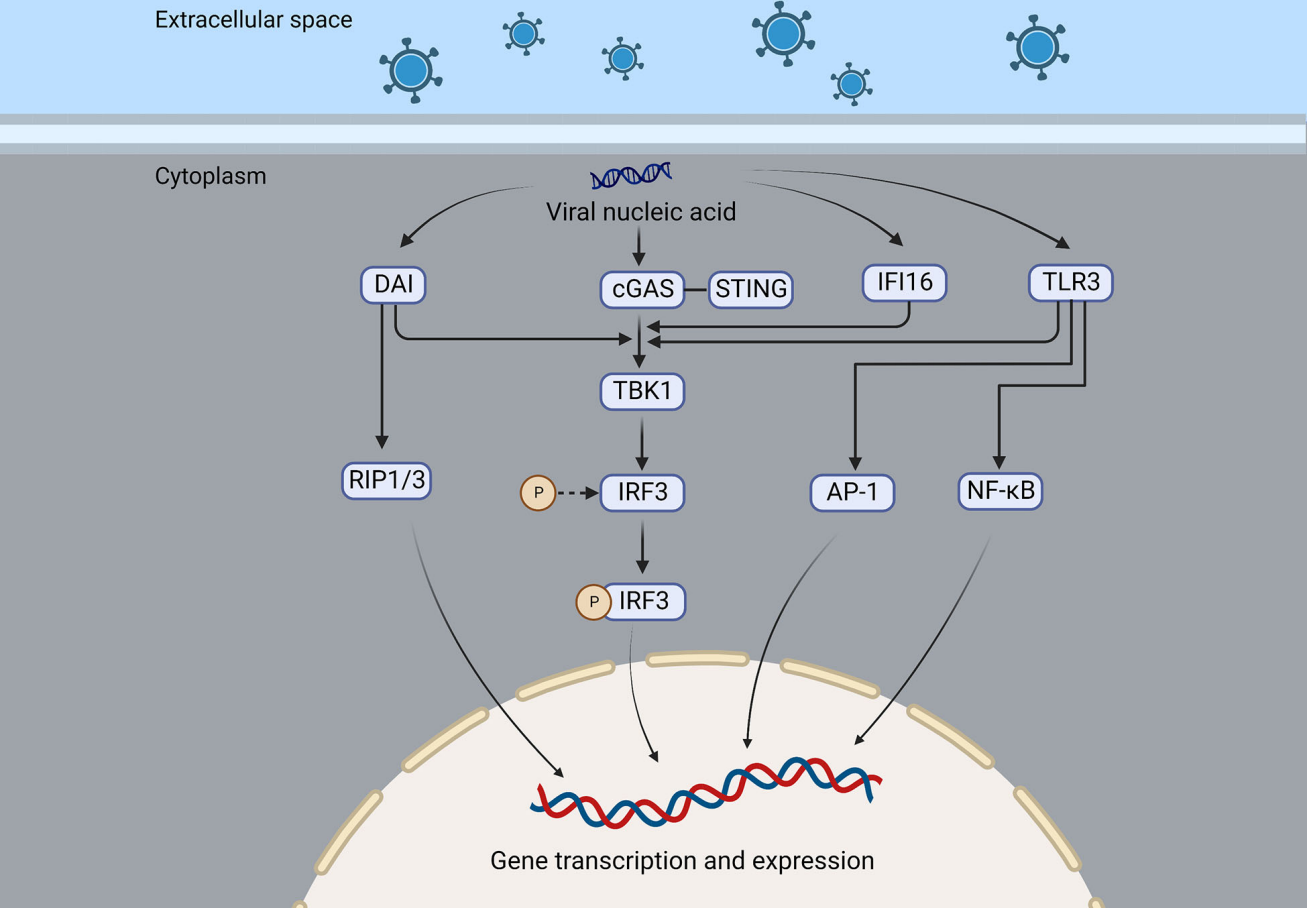
DAI, cGAS, IFI16, and TLR3 are nucleic acid sensing mechanisms of the virus that recruit and activate TBK1, which in turn activates IRF3, resulting in the expression of interferon or cytokines. In addition, DAI can also recruit RIP1/3 to promote gene transcription and expression, and TLR3 can activate NF-κB and AP-1 and induce the production of interferon and inflammatory factors.

Also, Toll-like receptors (TLRs) play a crucial role in recognition of viruses by the host. HSVE pathogenesis is linked to TLR3 deficiency (46). In the TLR3 molecule, an ectodomain (ECD) is present inside the endosome, and an extracellular Toll/interleukin-1 receptor domain (TIR) is present outside the endosome. The ligand-binding ECD domain promotes the phosphorylation of TLR3, and the TIR domain recruits adaptor proteins, which are important for downstream signaling (47). Multiple cells express TLR3, which recognizes double-stranded RNA (dsRNA), an intermediate in viral replication (48). When TLR3 binds to its ligand, it recruits its only adaptor-TRIF (or TICAM1)-triggering downstream signaling that activates TBK1, an inhibitor of κB (IκB) kinase-related kinase-ϵ (IKK-ϵ), and phosphorylates IRF- 3, while phosphorylated IRF-3 is translocated to the nucleus to induce interferon gene transcription (49–52). TLR2, 7, 9, and other subtypes also contribute to viral recognition (53, 54) (**Figure 2**). UNC93B1 is a multi-transmembrane protein that plays a crucial role in necleic-acid-sensing TLR signaling (55). Studies have shown that the UNC93B1 regulates the TLR7/9 signaling pathway by transferring TLR7 and 9 to endolysosomes (56). UNC93B1 prevents the STING from hyperactivation, thus inhibiting the cGAS-STING pathway and its subsequent interferon production, this was shown in UNC93B1-deficient mice that UNC93B1 deficiency strengthens the host immune responses to the cytosolic DNA stimulation and UNC93B1-deficient mice are more resistant to HSV-1 infection (57).

### 3.2 Immune evasion

Even though the host has many antiviral mechanisms, the virus has developed a powerful immune evasion mechanism (58). The enzymatic activity of cGAS is crucial to antiviral effects by triggering downstream interferon signaling by binding to dsDNA. Interferon mRNA was significantly higher in VP22 knockout HSV-infected cells than in wild-type HSV-infected cells, and ectopic expression of viral proteins VP22 are shown to inhibit cGAS/STING-mediated interferon production (59). VP24 can inhibit cGAS and STING-induced promoter activation and interferon production (60).

Many studies have revealed that the tegument proteins are important in viral gene replication and assembly (61). Among them, UL36 ubiquitin-specific protease (UL36USP) acts as a deubiquitinase that inhibits promoter activation of interferon and NF-κB induced by cGAS and STING, which allows the virus to evade host DNA sensing immune responses (62). Furthermore, UL36USP inhibits the degradation of capsids due to its deubiquitylase activity and prevents the viral genome from entering the cytoplasm, thus preventing DNA sensing-induced antiviral immunity (63). UL24 is a conserved protein among the herpes family but essential for viral replication, it can inhibit interferon and interleukin-6 (IL-6) expression mediated by cGAS-STING, and UL24 is also found to block NF-κB promoter activation. All these lead to viral immune evasion (64). A deamidation of the viral tegument protein UL37 inhibits the synthesis of cGAMP catalyzed by cGAS, interrupts downstream signal transduction, reduces interferon production, and promotes viral survival (65). Even though the cGAS-STING pathway is essential for the host against the virus, HSV-1 has evolved to evade host immune responses. Compared to the UL41-null mutant virus, wild-type HSV-1 infection could inhibit activation of the interferon signaling pathway, and UL41 expression inhibits interferon promoter activation and decreases production (66).

ICP34.5 is the virulence factor of HSV, encoded by a leaky-late gene, which can dephosphorylate eIF2α under the action of protein phosphatase 1-α, thereby allowing the continuous synthesis of viral proteins (67). ICP34.5 inhibits downstream antiviral signaling by preventing STING’s translocation to the Golgi apparatus (68). IFI16 can induce interferon production early in infection and exert antiviral effects (69), whereas later in infection, ICP0 targets IFI16 for degradation through its E3 ubiquitin ligase activity and promote virus replication (70). β-catenin is crucial for regulating the transcription of target genes. However, HSV-1 US3 protein inhibits interferon production by phosphorylating β-catenin in the Wnt signaling pathway and further restricting β-catenin nuclear translocation, thus antagonizing the interferon production and destroying the host antiviral immune response (71).

### 3.3 Immune response in HSVE relapse

Infectious, autoimmune, and postinfectious encephalitis is the most common causes of encephalitis, characterized by inflammation of the brain parenchyma with neurological deficits (1), with viral encephalitis accounting for 60% of infectious cases (72). HSVE is one of the most common causes of encephalitis, and although the virus is cleared after regular treatment, patients still experience relapses in neurological symptoms. In some patients, viral DNA was detected in their cerebrospinal fluid, indicating persistent infection or viral reactivation, which signified a true relapse of HSVE.

In some patients, however, the virus was not detected by cerebrospinal fluid PCR after relapse, and the condition improved after Immunotherapy (73), suggesting that the immune mechanism lies at the heart of many of these complications. The authors reviewed a total of 43 patients with herpes simplex encephalitis and anti-N-methyl-D-aspartate receptor (NMDAR) antibody encephalitis, most of whom were children with a biphasic course (74), and anti-NMDAR antibody encephalitis is the most common immune encephalitis after HSVE (75). Additionally, anti-GABAR antibodies, anti-CASPR2 antibodies, and some unknown antibodies will be produced after HSVE (76–79). 27% (14 of 51) of HSVE patients had autoimmune encephalitis (AEs), and all 14 had neuronal antibodies, while 11 of 37 patients without AEs also had neuronal antibodies (80). It was assumed that the viral infection triggered the immune response because none of the patients had these antibodies before developing HSVE. When mice were intranasally injected with HSV-1, Linnoila et al. found that serum NMDAR antibodies were positive, hippocampal NMDAR decreased, and also produced unknown antibodies (81), which had been observed in patients with autoimmune brains after HSVE.

When combined with anti-NMDAR encephalitis (following a non-HSV infection), patients are more likely to develop HSV antibodies than controls (compared with Cytomegalovirus and Epstein-Barr virus), and there are no neuronal or glial markers in the CSF, it is considered that HSV and NMDAR might be connected (82). A molecular mimicry best demonstrates the link between Campylobacter jejuni and Guillain-Barré syndrome (83). Zhao et al. found that, compared with wild-type HSV-1, the virus with protein UL6 gene knockout could not induce autoimmune diseases and that wild-type autoimmune diseases were triggered by autoreactive T cells (84), suggesting that molecular mimicry may contribute to autoimmune disease development following viral infections.

Despite this, molecular modeling alone may not be sufficient to explain immune encephalitis since HSVE is often associated with extensive neuronal damage, leading to the release of antigens from neurons (73, 74, 85), the presence of unknown antibodies could also explain symptoms other than typical NMDAR encephalitis (86, 87). Previously, 33% of patients with anti-NMDAR encephalitis had abnormal brain MRIs, but few had contrast enhancement (88), while most patients with autoimmune encephalitis after herpes simplex encephalitis had contrast enhancement, suggesting disruption of the blood-brain barrier (89) or inflammation (80, 90).

**Figure 3:**
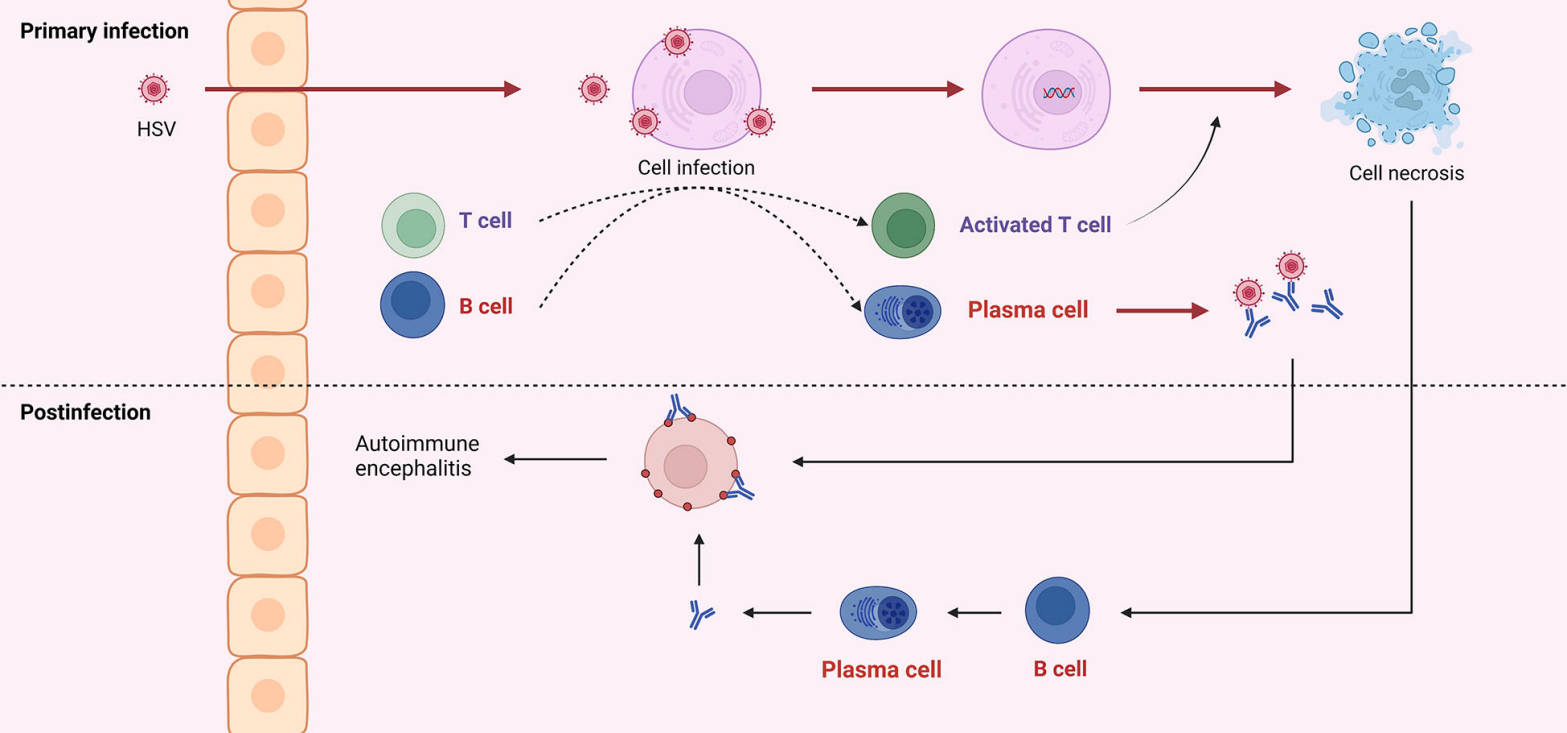
HSV into the central nervous system can cause infection of neurons, which is referred to as primary infection. Both viral particles and infected cells can recruit B and T cells. Antibodies produced by B cells can neutralize virus particles, and T cells can exert cytotoxicity to kill infected cells. Due to the similar structure of viral surface antigens to self-tissue, antibodies derived from the primary infection may attack healthy neurons. And cell disintegration leads to self-antigen exposure, induces B cells to produce antibodies, and further attacks self-cells, which is called post-infectious encephalitis or autoimmune encephalitis.

According to Omae et al., the cerebrospinal fluid cytokines or chemokines in patients with NMDAR encephalitis after HSVE increased in the early stage, suggesting immune infiltration into the central nervous system and damage to blood-brain barrier integrity. After treatment, these cytokines or chemokines gradually decreased; then, in the middle stage, they increased again, but NMDAR antibodies were absent; finally, in the late stage, NMDAR antibodies reached their peak, and the cytokines and chemokines gradually decreased (91). In an evaluation of this case, Wesselingh et al. proposed a hypothesis of the pathogenesis of autoimmune brain following herpes simplex encephalitis: HSV infection results in a breach of the blood-brain barrier that allows innate/adaptive immune cells to infiltrate and cause neuroinflammation. Eventually, B cells and T cells are recruited and produce antibodies against neuronal antigens (90, 92, 93) (**Figure 3**).

## 4 The efficacy and prognosis of standardized Immunotherapy

Patients with herpes simplex encephalitis experience a variety of clinical symptoms. The most common symptoms are headache, fever, and focal neurological symptoms. In severe cases, there may be unconsciousness (94). Early identification and targeted treatment are of great significance to the prognosis of patients.

Aciclovir is a nucleoside analog with potent antiviral properties against herpesviruses. As the first-choice treatment for HSVE, acyclovir has been proven in two previous randomized controlled trials (95, 96). Infectious Diseases Society of America, clinical practice guidelines, recommend treating patients with suspected encephalitis empirically with acyclovir before diagnosis, and for the specific treatment of HSV, acyclovir is also a class III recommendation (97). The British Association of Neurologists and the British Association of Infectious Diseases recommend that if there are no clinical contraindications, cerebrospinal fluid pressure, white blood cell count and classification, protein, and sugar be collected as soon as possible after admission. If there are contraindications, a head CT scan should be performed as soon as possible. When cerebrospinal fluid or imaging suggests viral encephalitis, acyclovir antiviral therapy should be started (10 mg/kg, tid, 14-21 d) (98).

The role of corticosteroids in HSVE is not yet clear, but the treatment is expected to improve cerebral edema, high intracranial pressure, and structural displacements of the brain empirically. In theory, corticosteroids could exacerbate the illness by promoting viral replication. In studies of mice treated with acyclovir combined with corticosteroids, however, the viral load in the brains of mice treated with acyclovir alone did not change significantly, and brain MRI abnormalities in mice treated with corticosteroids decreased significantly (99), demonstrating that corticosteroids can benefit brain injury without affecting viral loads. Acyclovir plus dexamethasone or dexamethasone alone reduced viral load compared to controls (100). A retrospective study describes the benefits of concomitant corticosteroids in patients with HSVE (101). However, British guidelines advise against routinely using corticosteroids for treatment, possibly due to their side effects (98). Corticosteroids have not yet been determined to be the most effective treatment for HSVE. However, if there is obvious edema or mass effect, it is recommended to continue corticosteroids (102). However, animal studies have shown that delayed corticosteroid addition suppresses inflammation and viral genes (103).

In addition to seizures, movement disorders, psychosis, and cognitive changes, NMDAR encephalitis may occur sometime after HSVE (104). The study by Nosadini et al. found that dyskinesia is one of the key symptoms to distinguish HSVE-induced AEs from pure recurrence of HSVE (74). A comprehensive etiology and imaging examination should be performed if symptoms recur and it is impossible to differentiate between virus reactivation and immune induction. New hemorrhage or necrosis on brain MRI often indicates viral replication (74). It is important to consider autoimmune encephalitis if viral testing is negative and to initiate Immunotherapy as soon as possible (105). Immunotherapy is effective in several studies (90, 106–108). AE after HSVE is treated similarly to NMDAR encephalitis, with plasma exchange, corticosteroids, immunoglobulin as first-line treatments, and immunosuppressants, including rituximab, as second-line treatments (109). According to a study, half of the patients with NMDAR encephalitis gradually improved after receiving first-line treatment within 4 weeks. The remaining patients who did not respond well to first-line treatment received a second-line treatment, which was more effective than no treatment (88).

## 5 Conclusion

It is a common infectious encephalitis caused by HSV. Although many studies have revealed its etiological mechanism, and many targeted treatment options have been developed, the prognosis is still unsatisfactory, especially for HSVE. The immune system plays an important role in the pathogenesis of herpes simplex virus encephalitis, which is also why corticosteroids play an important role in treating autoimmune encephalitis. Whether it is a molecular simulation or neuron damage, the speculation about the pathogenesis of immune encephalitis is constantly being confirmed. Immunotherapy can have certain curative effects, but the timing of initiation of Immunotherapy is still uncertain, and further research is necessary. In this article, we reviewed the general disease characteristics of herpes simplex virus encephalitis, summarized potential immune mechanisms, and discussed its important complication, autoimmune encephalitis, in the hopes of providing further insight for future research.

## Author contributions

LinZ, LijZ, LF, LW, JY and HZ designed and wrote the manuscript. JT, CY and ZX helped with proofreading and revision. All authors contributed to the article and approved the final version.
